# The “Immigrant Medical Services” Organization from the End of the British Mandate Through the First Years of Israel (1944–1953)

**DOI:** 10.5041/RMMJ.10541

**Published:** 2025-01-30

**Authors:** Dorit Weiss

**Affiliations:** Health Administration, Netanya Academic College, Netanya, Israel

**Keywords:** American Jewish Joint Distribution Committee, Hadassah, health policy, immigration, medical services for immigrants, medicine history

## Abstract

The aftermath of the Second World War and the Holocaust triggered mass migration of Jewish refugees to British Mandatory Palestine and, after 1948, the nascent State of Israel. Responding to this crisis, Jews in the Diaspora increased their commitment to facilitate immigration to Israel, particularly by supporting medical services to the Yishuv (pre-state Jewish Settlement). This paper explores the critical role played by Hadassah and other organizations in establishing direct medical services for Jewish immigrants during two key periods of Israel’s history: the end of British Mandatory Palestine (1944–1948) and the early years of the State of Israel (1948–1953). While the Immigrant Medical Services organization faced numerous challenges, this organization was essential in addressing the pressing healthcare needs of a burgeoning population amid morbidity and mortality concerns. An emphasis is placed on the challenges faced by these organizations and the commitment and resourcefulness of all involved, which ultimately shaped the foundation of Israel’s healthcare infrastructure.

## INTRODUCTION

The Second World War (WWII) and the Holocaust led to the migration of millions of Jewish refugees and displaced persons across countries and continents. After the war, many began to immigrate to British Mandatory Palestine and (after May 1948) the State of Israel. This led to an intensified commitment from Diaspora Jews to aid immigration to the *Yishuv* (Jewish community and its organization in pre-state Israel; see Glossary for details) in British Mandatory Palestine, expressed primarily through economic and humanitarian aid, including medical services.

Two American organizations were involved in providing medical aid to the Yishuv. The first was the American Jewish humanitarian organization Hadassah: The Women’s Zionist Organization of America (hereinafter, Hadassah), particularly prominent in the establishment of direct medical services for the Yishuv, unlike most other Jewish organizations that only provided assistance and support from abroad. From its establishment, one of Hadassah’s goals was to expand the Yishuv’s healthcare capabilities with a foundation for the future. Hence, it was natural that, when asked, Hadassah would become involved with the management of the Immigrant Medical Services (IMS), thereby effectively implementing Hadassah’s mission. The second was the American Jewish Joint Distribution Committee (JDC), which began providing direct medical care after the establishment of the State of Israel.

This paper provides an overview of the medical services provided to Jewish immigrants between 1944 and 1953, comprising the end of British Mandatory Palestine and the early years of the State of Israel, with a focus on two key issues. The first relates to establishment of the IMS as a solution for absorbing the Jewish immigrants amid fears of morbidity due to infectious diseases and mortality. The second refers to the vital assistance provided by Hadassah in the midst of mass immigration to Israel. Hadassah helped bridge the growing gap between immigrant needs and available healthcare services. Israel’s present healthcare system, which is successfully serving a diverse, multicultural citizenry of more than 9 million people, owes its success first and foremost to the few individuals who, through improvisation and sacrifice, did their utmost to enhance the health of the nation’s immigrant population. A glossary is provided herein, for the convenience of the reader.

## OVERVIEW: HEALTHCARE AND IMMIGRATION IN PRE- AND POST-STATE ISRAEL

### Healthcare Prior to the British Mandate

Healthcare was dire in pre-state Israel under the Turkish Ottoman Empire (also referred to as Ottoman Palestine) following the end of the First World War (WWI), and there was a lack of any real sanitary infrastructures. Until 1914, multiple diseases, such as malaria, trachoma, cholera, and typhus, were widespread in the Jewish Yishuv, reaching epidemic proportions. Having only limited capabilities, the small Jewish presence in pre-state Israel was helpless in confronting these outbreaks. A few physicians worked in the Christian Mission hospitals and Jewish charity institutions, but their resources were limited. Only a handful of registered nurses were in the land at the time, mainly nuns at Christian Mission hospitals responsible for overseeing patient care.

The founding of Hadassah in New York in 1912, following Henrietta Szold’s visit to Ottoman Palestine with her mother in 1909, marked a turning point for Jewish healthcare. Under her leadership, Hadassah would play a critical role in advancing healthcare and social welfare in pre- and post-state Israel. Hadassah’s role is discussed in more detail below.

Several other organizations in Israel were established at this time, all of which provided essential services to the Yishuv. *Kupat Holim Clalit* (Clalit Health Services), an early form of health maintenance organization (HMO), was established in 1911, and the Magen David Adom ambulance service was founded in 1919.

The American Jewish Joint Distribution Committee (JDC) was founded in 1914 to assist European Jews. They would become the main body to fund Jewish immigration to pre- and post-state Israel during WWII and the subsequent decade.^1^,^2^

As a result of the aid from these organizations, healthcare in the Yishuv continued to improve. Concomitantly, the state of health of the Arab population also improved compared to that of other neighboring Arab countries. Some Arabs in Mandatory Palestine also benefited from the Jewish healthcare services; they were admitted to the Hadassah hospitals and the Clalit Health Services clinics for humanitarian and political reasons.

Under the British Mandate, the Healthcare Department of the Jewish National Council (JNC) coordinated all Jewish healthcare services in the Yishuv. However, they lacked the authority and facilities to influence and direct healthcare policy. Hence, Hadassah and Clalit had a notable influence via their respective organizational bases—Hadassah through its connection to the United States, and Clalit with the support of a new labor organization established in Mandatory Palestine in December of 1920, namely the Histadrut.

### Healthcare Under the British Mandate

The start of British rule on July 1, 1920 marked the end of martial law in the country and spurred accelerated development in pre-state Israel.

Under the British Mandate, Jewish medical services to the Yishuv were mainly provided by Clalit Health Services. Those services would extend into Israel’s early years. From 1920, Clalit provided health insurance to Jewish workers in the Yishuv and a network of clinics and hospitals. There were other Jewish HMOs as well, but they were small with limited activities.^3^ The British Mandatory Government also provided medical services to the Yishuv and Arab population until Israel’s independence in 1948.

Mandatory Palestine was unique among the British colonies due to its religious-historical value and its importance to the British people. The Arab–Jewish conflict further heightened interest in developments there. Additionally, Zionist institutions were actively working to garner political support in Britain, pressing the issue of the future of the British Mandate in Palestine above other challenges facing Britain’s colonies. From the outset, administering British Mandatory Palestine required a carefully considered and balanced policy that, in actuality, would be challenging to implement.

Jewish immigration (*aliyah*) and immigration quotas for Jews were key issues of concern to the British and the Yishuv even before WWII. With the rise of Nazism, Jews were already fleeing Germany, leading to an increased number of Jewish immigrants, from 31,500 in 1934 to 62,000 in 1936. Following establishment of the State of Israel in 1948, the immigrants’ health became a critical factor in the context of mass immigration and medical screening. The engagement of both Hadassah and the JDC in intensive efforts to fund and operate immigrant services was a key factor enabling the nascent State to maintain an immigration policy during this period of mass immigration, including absorbing many sick, disabled, and elderly.

Under the British Mandate, the basic infrastructure that would serve the new Israeli health system was constructed, including improvements in the water supply, sanitation, and disease prevention. During WWII, the British issued healthcare regulations for registering births and deaths, guidelines for pharmacists and midwives, and regulations for reporting infectious diseases and vaccinating babies against smallpox.^4^

### Healthcare in the Nascent State of Israel

Establishment of the State of Israel on May 14, 1948 spurred an unprecedented wave of Jewish immigrants in the history of the Zionist movement. From the Declaration of Independence to the close of 1951, about 700,000 immigrants arrived in Israel, doubling the country’s Jewish population.^5^

Immigration during this period emanated primarily from the Balkans, Eastern Europe, and countries in Asia and North Africa. The total number of Holocaust survivors and WWII refugees—about 330,000 persons—constituted approximately half the immigrants during this period. Around 370,000 immigrants came from Asia and North Africa; roughly 45,400 came from North Africa while the rest came from Asia, including 123,300 from Iraq, 48,300 from Yemen, and 34,500 from Turkey.^5^

By the end of 1951, more than half the immigrants were of Asian and African origins, whereas immigration during the British Mandate (1918–1948) was composed primarily of persons of European and American descent. Pressing external and internal political pressures necessitated rapid organization and absorption of the immigrants. The situation made it almost impossible for them to sell their possessions, liquidate their businesses, or even prepare themselves emotionally for life in a new country—preparations that presumably could have eased their absorption. Israel became, primarily, an immigrant nation. Notably, while the Jewish population in 1948 consisted mainly of immigrants, by the end of 1951 the percentage of immigrants remained quite high—75% of the Jewish population. The majority of immigrants arriving in Israel were destitute and suffered from very poor health, including severe malnutrition. Physically and mentally exhausted, they experienced a high incidence of morbidities such as tuberculosis, ringworm, and trachoma, particularly children and the elderly. Furthermore, a high percentage of them were disabled, extremely frail, or mentally ill; all of whom were unable to care for themselves, let alone navigate the absorption process in an unfamiliar country. Among the Jews who immigrated from Arab and North African states, approximately 40% suffered from tuberculosis and skin, eye, and kidney conditions. Immigrant children suffered from weakness and rickets caused by malnutrition.^6^ According to reports of the JDC, 10% of the immigrants arriving in Israel suffered from diseases requiring immediate hospitalization. The State of Israel, however, lacked the hospital bed capacity to meet such needs, and most of the immigrants initially remained without appropriate care. This situation created a pathway for contagion with the danger of widening the circle of contagious diseases and posed an ongoing threat to the immigrants themselves and the wider public.7a

Moreover, conditions were harsh in the temporary housing offered in the transit camps (see Glossary for details); lacking elementary sanitary conditions, the camps saw infant mortality rise among the immigrant population. Furthermore, lack of transportation between the transit camps and hospitals contributed to 15% of infant deaths occurring at home during the first year of life. While in veteran communities, infant mortality stood at 16.2 deaths per 1,000 babies, infant deaths among immigrants in the transit camps surpassed 157.8 per 1,000.^8^

The prevalence of trachoma and ringworm among the immigrants stood at 10%, mostly among newcomers from Yemen and North Africa.^9^ In 1948, the death toll from tuberculosis in Israel averaged 230 persons annually; this rose to around 1,500 fatalities annually during 1952–1957.^10^ Data from medical checkups at the Shaar HaAliyah immigrant intake camp, located south of Haifa Port, indicated that 4% of the screened immigrants had tuberculosis, half of which were active cases. Ministry of Health forecasts for 1948–1951 estimated that 4,300 hospital beds would be needed to treat tuberculosis patients among the immigrants yet to arrive; however, the Ministry had only 1,975 hospital beds earmarked for TB patient care—less than half those needed.^11^

The new Israeli government established the Israeli Ministry of Health, which took responsibility for providing medical services and building hospitals using the infrastructure left by the British. They also took advantage of the IMS (discussed in more detail hereinafter), which had been established in 1944 by the Jewish National Council (JNC) and the Jewish Agency (the executive branch of the World Zionist Organization, see Glossary), and operated until 1953.7b

## HADASSAH’S ROLE IN PRE- AND POST-STATE ISRAEL

When Hadassah was established in 1912, the organization’s main objectives were two-fold: to promote Zionism, particularly in the US, and to develop healthcare services in the Land of Israel. A basic understanding of Hadassah’s role in pre- and post-state Israel clarifies why the organization’s involvement was so critical to the IMS.

It merits noting that Hadassah benefited from the growth of women’s rights organizations. American Zionist women were seeking platforms for public social work. Although Zionist organizations would not accept their help prior to the First World War, after the war it was becoming more acceptable.^12^ Most female Jewish activists came from a middle-class background, and all had benefited from higher education. Focusing their activities on the pre-state Jewish Yishuv was a convenient channel for them compared with the struggles they would have had to undergo in the USA.

Initially, Hadassah focused on public health and midwifery. Their first delegation, two registered nurses, came to Ottoman Palestine in 1913. With the outbreak of WWI, one of the nurses went to help treat Jewish exiles in Egypt.^13^ More delegations with expanding responsibilities would follow.^14^ By 1944, the organization was impacting healthcare of the entire Jewish community in the region.

The organization found itself becoming increasingly involved in healthcare for Jewish immigrants. This is, perhaps, partly due to the leadership of Dr Haim Yassky, the medical director of Hadassah. He had anticipated a mass immigration to and its impact on pre-state Israel, well before WWII. In 1944, he presented a 12-page plan, comprehensive in scope, to the Yishuv. His vision and commitment were essential to the local implementation of Hadassah’s healthcare goals for the Jewish community. Eventually, Hadassah would take over management of the IMS (discussed in more detail below) and appointed Yassky as its director.

The organization appeared to officially change focus in the winter of 1946 when it passed a resolution to provide healthcare services to all European refugees upon entry to pre-state Israel. From this point on, Hadassah assumed the treatment of the new immigrants, focusing on medical checkups, urgent hospitalizations, tuberculosis and infectious disease screenings, and nutrition and medical follow-ups.^15^ The same year, Hadassah saw more than 2,000 children vaccinated against diphtheria and arranged for the shipment of advanced medical equipment to pre-state Israel.^16^ In May of 1946, when it began to appear that large-scale immigration would become a reality, Yassky kept Hadassah’s directors in Jerusalem apprised of the anticipated medical challenges and suggested establishing a collaborative effort between all the medical entities in pre-state Israel.^17^

The next two years would see a dynamic interplay between humanitarian initiatives, healthcare challenges, and the political realities of the time as the Yishuv sought to overcome the Holocaust’s aftermath and lay the foundation for a future state. Hadassah had become highly respected and was considered a leader from which the Yishuv could learn.^18^ Financial resources would remain challenging,^19^,^20^ although Hadassah continued to support medical care as much as possible via donations^21^–^23^ and establishing new healthcare institutions led by Hadassah medical staff^24^—despite the resulting strain placed on the hospital in Jerusalem.^25^

From the 1950s onward, Hadassah would gradually cut back its activity and focus on a new hospital that opened in Ein Kerem. In line with their ideology to develop sustainable local healthcare, many of the clinics they opened would be transferred to external entities. However, to this day, Hadassah remains a major player in Israeli healthcare.

## THE IMMIGRANT MEDICAL SERVICES ORGANIZATION 1944–1953

As can be seen from the above historical background, a mass immigration to pre- and post-state Israel was inevitable. Clearly, these immigrants would need immediate medical care both in their countries of origin and at their destination point. This need ultimately led to establishment of the Immigrant Medical Services (IMS).

### The Challenges of Mass Immigration

The wave of immigration that began in 1944 saw many women and children fleeing Europe, with fewer working-age men. Most immigrants lacked professional skills and were in poor health, suffering from depression, malnutrition, and chronic illnesses. Many were incapable of working. Some 40% of immigrants arriving from Asia had tuberculosis, skin diseases, eye and kidney diseases, and other ailments, while many children suffered from dystrophy, atrophy, and rickets.^26^–^28^

To address these challenges, the Medical Development Committee, operating on behalf of the JNC, which represented the needs of the Yishuv, recommended expanding existing Yishuv medical and social services in collaboration with the Jewish Agency’s Immigrant Absorption Department. They proposed that medical stations be established in immigrant countries of origin that had inadequate healthcare services. Each station would be staffed by a physician and a nurse. Medical personnel would accompany the immigrants during their journey to Mandatory Palestine. Upon arrival the immigrants would initially be housed in camps adjacent to the port and receive initial medical and social care and be registered with an HMO. There would also be facilities for patient care, recuperation, and nurse-supervised childcare. The camps would house up to 500 people for up to 4 weeks and be fully disinfected between groups of immigrants.

### Establishment and Management of the IMS

As mentioned above, in June 1944 Dr Yassky, who had anticipated this mass immigration, presented a comprehensive plan that anticipated the end of WWII and the healthcare needs of the Yishuv. Estimating the scope of the anticipated mass immigration was impossible; hence, his plan detailed the needs for rural districts, towns, and hospitals throughout the country. Addressing three areas of medical need—prevention, curative treatment, and medical staff education—he recommended provision of specific services by different bodies: ambulatory services by the HMOs, preventive medicine by Hadassah, and rehabilitation by the Jewish Agency and the Jewish National Council. A combination of the Mandatory government, HMOs, and Hadassah would meet hospitalization needs, with the British Mandatory government being responsible for care of the mentally ill.^29^ To improve relations with the Mandatory government, Yassky also recommended establishing an advisory health council for the government’s Healthcare Department that would include representation for the Yishuv’s medical institutions.^30^

In considering the absorption of these specific immigrants, the Yishuv feared morbidity and the spread of infectious diseases; there were also concerns regarding medical screening, entry examinations, and medical insurance. After much planning and discussion, the JNC and its Healthcare Department established the IMS in late 1944; logistically, the IMS would operate under the auspices of the Jewish Agency, since it was tasked with management of the healthcare needs of the Jewish immigrants with the assistance of various different healthcare institutions, including Hadassah, Kupat Holim Clalit, the Magen David Adom ambulance service, the Women’s International Zionist Organization (WIZO), and various local hospitals. Dr Theodor Grushka from Hadassah was appointed Medical Director and Supervisor of the IMS.^31^,^32^

The JNC established a public committee to discuss immigrant healthcare, initially focusing on the IMS budget. However, the public nature of the committee led to prolonged discussions without significant progress in establishing the needed healthcare facilities. Hence, when Grushka was eventually appointed as physician in charge, he lacked administrative support. Dr Katznelson, representing the JNC and serving as committee chairman, was also criticized for not opening much-needed maternity care and tuberculosis facilities, but had been impeded by the ongoing meetings. Eventually, the committee was disbanded and only a great deal of background involvement from others assured financing of the IMS with Grushka as its director.^33^

### Hadassah’s Role in Managing the IMS

The IMS began operating under Grushka’s leadership, and a plan was developed to provide Jewish immigrants with free access to hospitalization in Hadassah Hospital for a period of 6 months.^32^ However, by the end of 1945, the medical services to Jewish immigrants had not been significantly changed. Already noting the lack of progress, the JNC asked Hadassah to consider collaborating with and funding the IMS, and, in parallel, the Jewish Agency asked Hadassah to increase its share of funding it. Hadassah proposed taking over management of the IMS, believing that the Jewish Agency would finance half the cost if Hadassah agreed to do the same.^34^

Discussions could not help the IMS achieve its goals, and Grushka, lacking authority, staff, and budget to develop adequate health services, resigned in July 1945; when asked, he pressed on with the work. In September, Grushka met with the Jewish Agency’s Aliyah Department and submitted a proposal for continuing the activities of the IMS. However, Grushka resigned a month later, frustrated by the ongoing lack of improvement to the situation.^35^

Although Hadassah’s management had already approved an urgent proposal to take over management of the IMS in early January, the decision was only formalized on October 1, 1946.^36^ Once in official management of the IMS, Hadassah reinstated Grushka as director.

The formal agreement was signed by the Jewish Agency, Hadassah, and the JNC. This was clearly a wise decision, as Hadassah was already well positioned to meet new immigrants’ medical needs under Yassky’s leadership. At the signing ceremony, Yassky stressed the importance of cooperation between all the institutions involved, if they were to succeed in providing comprehensive medical and mental healthcare to the immigrants.^37^

The intervening months, particularly from June until October 1946, had been spent recruiting and negotiating with all the involved institutions, assessing medical needs, and developing a comprehensive plan to ensure effective healthcare services. The JDC was recruited to address the challengingly high number of disabled Jews in the displaced person camps; working together with UNRRA, the JDC would bring the Jewish immigrants to British Mandatory Palestine.^38^ In June 1946, an agreement was signed that outlined Hadassah’s responsibilities in managing the IMS. Hadassah was tasked with examining immigrants only upon their arrival in British Mandatory Palestine (the Jewish Agency would be responsible for immigrant medical examinations abroad). This included providing medical services in immigrant housing and transit camps, general and specialized hospitalization, convalescence, medical equipment supplies, dental care, and preventive medicine. The IMS would meet the immigrants’ medical needs for one year, with specific exceptions. Hadassah was authorized to collect fees from patients and their families to help cover the costs of medical services; fees would be determined by the HMOs on a sliding scale. Furthermore, the British Mandatory government would also allocate funds to the IMS.^39^

### 1946–1947: Ongoing Challenges

The continuous influx of Jewish immigration presented a variety of medical problems that complicated Hadassah’s original plans in managing the IMS. The enforcement of strict immigration quotas (1,500 per month) and deportation of illegal immigrants by the British Mandatory government presented further challenges, and IMS activities were limited.^40^ Despite the quota, more illegal immigrants were entering the country than legal ones.^41^ Hence, the British Department of Health closely monitored the Jewish immigrants and their health status.^42^

During the first IMS management meeting in 1946, plans were developed to expand the housing and medical facilities for the immigrants.^43^,^44^ However, Hadassah quickly realized that they had underestimated the costs for managing the IMS. Preliminary estimates of 2,500 Palestine Pounds (£P) per month per person were markedly lower than the actual expenditure of £P9,600. Adding to the financial strain was the cost of maintaining a hospital in the Atlit detention camp^45^ and investments to expand buildings and infrastructure.^46^

Establishment of the IMS led to a complex reassigning of roles and responsibilities for immigrant healthcare. The IMS was required to perform a physical examination on each immigrant before providing medical care. Hadassah provided all the health care services, including the examinations, to immigrants in the camps; immigrants sent directly to permanent housing were examined by a local HMO physician. Immigrants who failed to undergo this exam within one month of their arrival were not entitled to HMO services. After selecting an HMO, the Jewish Agency funded the immigrants’ insurance for the first three months after leaving the camps. Sick immigrants and women in labor were not charged for hospital admissions. Conversely, immigrants with severe conditions such as tuberculosis and mental illness were disqualified from joining an HMO; their care was funded by the Jewish Agency.

In addition to healthcare in the camps, other IMS services included emergency dental treatment and preventive care. This was, in fact, Israel’s first “medical services basket,” managed and controlled by Hadassah.^47^ But the available budget was insufficient to care for patients with chronic conditions.^48^ There was a severe shortage of hospital beds, particularly for chronic conditions like tuberculosis.^49^–^51^ At least an additional 100–200 nurses were required to care for patients. However, despite plans to meet these and other needs, the request to increase IMS’s budget was denied,^52^ and key projects were frozen^53^ and eventually cut by the end of 1947, negatively impacting the ability to care for the immigrants. But many Jewish immigrants who had been hospitalized while living in the camps or immigrant housing had exhausted their medical insurance and were entirely dependent on the services provided by the IMS.^54^

By the end of 1947, facing the end of the British Mandate and the partition of its territory between Jews and Arabs, the IMS faced a new immigration challenge for which the Yishuv was completely unprepared. Medical staff in the Jewish illegal immigrant camps in Cyprus warned of a shortage of hospital beds and questioned the country’s readiness to receive patients, as did staff in the displaced persons camps in Germany.^51^

Hadassah Hospital on Mount Scopus (Jerusalem) advised they would need a budget increase of £P650,000 in order to accommodate this new wave of immigration.^55^ As 1947 drew to a close, the financial state of the IMS worsened. Safety concerns on the eve of the 1948 Arab–Israeli War made it impossible for the relevant bodies to convene a meeting and resolve the difficult situation. Hadassah was forced to cover the IMS’s additional budget deficits.^56^,^57^

### The First Years of the State of Israel (1948–1953)

Following the announcement of a proposed partition plan for Palestine by the United Nations General Assembly in November 1947, Hadassah prepared an operational plan for deployment after the establishment of the State of Israel.^58^

In the meantime, the IMS had already opened five new immigration camps to receive the anticipated 8,000 immigrants from the Cyprus detention camps, despite lacking the budget to do so.^59^ After Israeli independence in 1948, the IMS operated clinics and health services in 21 immigrant camps. However, it struggled with a severely depleted workforce and increasing hospitalization requirements.^60^ Camp medical services included administering smallpox and typhoid fever vaccinations, infectious disease testing, disinfecting immigrants, isolating patients with contagious diseases, and performing blood tests and chest X-rays.^61^ New immigrants unable to go through the regular immigration process because of their complex conditions or disabilities desperately needed special services that required additional funding. Between October 1947 and January 1948, the IMS budget deficit was £P2,500, owing to the unexpected mass immigration.

The question of the immigration of European Jews, many of whom were Holocaust survivors with severe illnesses, was first raised in 1947 when the British government announced its departure date from Mandatory Palestine: May 15, 1948. The timing was significant, since the future Israeli government was in its early planning stages, including the Ministry of Health, which would have to address healthcare for the incoming immigrants.

Hadassah’s organizers were not untouched by the situation. They were well aware of Yassky’s perspective from an opening address to the IMS in February of 1948. Among other comments, he pointed out that the IMS was completely unready to take in the new immigrants due to a substantial failure of financial and organizational support.^62^ The organization responded, and in May, 1948 Hadassah approved a three million dollar increase to that year’s budget, enabling increased involvement in providing medical services after Israeli independence.^58^

Further compounding issues were the escalating tensions in Mandatory Palestine. By March 1948, Jerusalem was intermittently cut off from the coastal plain region due to attacks by Arab militias during the lead-up to the 1948 Arab–Israeli War. Travel between Hadassah Hospital on Mount Scopus and the Jewish-controlled sector of Jerusalem was perilous, as the area was surrounded and under constant threat. This also made it difficult to transfer patients to Hadassah Hospital.^63^ Wounded Jewish soldiers occupied most of the hospital beds, further straining available medical resources.

The war made it difficult to absorb immigrants, and the IMS found it challenging to make appropriate assessments and preparation.^64^ Faced with the IMS’s increased needs, Hadassah felt that it had reached the end of its financial capabilities and considered two options: continuing to manage the IMS provided the Jewish Agency committed to covering its high expenses, or bow out. Hadassah feared that further budgetary diversions to the IMS would jeopardize emergency health services at Hadassah Hospital and paralyze its activities.^65^ In early April 1948, Yassky informed the Jewish Agency that Hadassah was reducing its IMS funding to £P80,000 per year.^66^ These were Yassky’s final days.^67^ On April 13, 1948, Arab soldiers ambushed a humanitarian medical convoy making its way to Hadassah Hospital on Mount Scopus, killing 78 people, including Yassky.

Having received no funds by mid-September 1948, Hadassah formally declared that it would no longer be financially responsible for the IMS.^68^ The nascent Israeli Ministry of Health, preoccupied with healthcare to those wounded in the war, requested Hadassah to continue managing the IMS, at least to the end of the year. Hadassah acquiesced, provided that the Jewish Agency financed any expenses that exceeded the allocated budget.^65^

In July 1949, the Israeli government signed an agreement with Hadassah; Hadassah would continue to manage the IMS, but the new Israeli Ministry of Health would finance any budgetary shortfall.^69^ However, on May 13, 1949, the Israeli government announced that the Jewish Agency, not the government, would fund the IMS. Support for the IMS was right back where it had started. In light of this jockeying for support, Hadassah’s ongoing management of the IMS until 1951 is admirable.

In April 1949, Israel’s immigration camps housed approximately 50,000 Jews, and their population was increasing daily. Closure of the displaced person camps in Europe forced Israel to accelerate immigration of sick Jews. By the end of November 1949, some 700,000 Jews had immigrated to the fledgling State of Israel.

Between 1949 and 1950, the magnitude of the expected immigration required an extra 3,600 general hospital beds and a similar number of specialist beds for patients with tuberculosis, mental illnesses, and disabilities.^49^,^50^

It was fortuitous that the JDC was seeking a new mission around this time. Learning of the need, they focused on creating a new organization to serve Jewish immigrants with disabilities.^70^ At the end of 1949, the cooperative efforts of the Jewish Agency, the Israeli government, and the JDC led to establishment of *Malben* (Hebrew acronym for Organization for the Care of Handicapped Immigrants) to care for immigrants with severe medical conditions.^71^,^72^ Establishment of Malben marked the beginning of the JDC’s operations in Israel.^72^ The JDC served as Malben’s manager until 1976, when its management was transferred to the Israeli government.^73^

### The Last Days of the IMS

The early 1950s saw a decline in the number of immigrants, and the IMS began to reduce its activity. Many of its employees left to work at Kupat Holim Clalit or the Ministry of Health, while others were either laid off or retired. However, the eventual closure of the IMS’s activities did not end the medical treatments or the arguments and discussions surrounding them.

Although the records regarding the total number of patients treated by the IMS were destroyed in a fire, it is known that after the establishment of the state, of the approximately 700,000 immigrants residing in Israel, 10% were ill and required hospitalization.^11^,^74^,^75^ Despite the differences in approach that emerged among those involved in managing the IMS, as well as the depleting resources and organizational difficulties, the IMS succeeded to provide sufficient healthcare services to thousands of immigrants in camps and transit centers, as well as to those arriving in Israel from the camps in Europe, Aden, Cyprus, and Mauritius.

## CONCLUSION

Despite economic hardship, until establishment of the State, the Yishuv felt obligated to provide healthcare to the Jewish community in Mandatory Palestine and in countries where the Jews had been living, mainly in Europe and refugee centers in transit to Mandatory Palestine. This included providing medical care for Holocaust survivors in the displaced persons camps across Europe as well as the illegal immigrants who had been deported by the Mandatory government and incarcerated in camps in Aden, Mauritius, and Cyprus as though they were prisoners of war, not to mention all of the legal and illegal Jews residing in Mandatory Palestine.

The Yishuv could not have provided these services without the aid of Jewish philanthropic and humanitarian organizations such as Hadassah (detailed herein) and the JDC. The Yishuv was initially ambivalent toward these American organizations. However, it also desperately needed the physical and economic support they offered.^76^ The motivations for providing the medical aid were not purely humanitarian. They also stemmed from the reality of the situation at that time, which must be viewed through the broader lens of the political, social, military, and healthcare contexts of that time.

Throughout its existence, the IMS had to contend with organizational bureaucracy, and complicated medical conditions amid a severe shortage of cash, equipment, and skilled human resources. Nevertheless, in hindsight, it is clear that its establishment was the *only* solution for the successful largescale absorption of Jewish immigrants and the challenge they placed on the healthcare system of pre- and post-state Israel. Only through a managed process, such as that offered by the IMS, was it possible to provide adequate medical care and ensure that Jewish immigrants could transition to become permanent residents with all the necessary medical certificates this entailed.

The complex state of healthcare services immediately following the establishment of the State of Israel led the Zionist leadership to ask that these services remain under the authority of the Jewish Agency. Eventually, the Ministry of Health was established by the young Israeli government, and management of the IMS was transferred to its authority. The IMS provided a foundation and infrastructure for the establishment of medical services in Jewish immigrant camps in the new State. Between 1948 and 1951, some 700,000 Jews immigrated to Israel—more than the total population of the pre-state Yishuv. Furthermore, aid from Hadassah, JDC, and other organizations enabled the nascent Israeli government to change its policy from selective to non-selective health immigration, thereby opening the doors to every Jew wishing to immigrate to Israel.^77^

Hence, the IMS and later Malben, under the management of Hadassah and the JDC, respectively, were foundational to the success of the Israeli health system. The contribution of the IMS was reflected by key public health indicators, including reduced infant mortality and eradication of epidemics.^4^ The organization also played a role in the creation of Israel’s current medical insurance system. In retrospect, it is clear that Israel owes an enormous debt of gratitude to those few medical professionals who did their best to ensure the public health of the Jews who immigrated to pre- and post-state Israel and, with the help of Hadassah and the JDC, helped secure the future of Israel’s healthcare system.

## GLOSSARY

*Aliyah*: commonly used Hebrew term for immigration.
*Jewish Agency*: established in 1929 as the executive arm of the World Zionist Organization, serving as the liaison with the Mandate government and, in practice, was the institution of Jewish governance
*Jewish National Council (JNC)*: the main national executive organ of the Assembly of Representatives of the Yishuv.
*Transit Camp*: temporary facilities set up by the British authorities to house Jewish immigrants arriving in Mandatory Palestine, often after fleeing persecution in Europe; after establishment of the State of Israel, the Israeli government also provided temporary facilities for all immigrants.
*Wave*: commonly used to describe the groups of people who immigrated to Israel; may be presented generally as waves, or specifically as, for example, “the wave of 1946” indicating all those immigrants fleeing the Holocaust in 1946.
*Yishuv*: commonly used Hebrew term referring to the Jewish community, its leadership, and organized efforts to establish institutions, develop infrastructure, and promote the Hebrew language and culture in pre-state Israel.

## Abbreviations

IMS: Immigrant Medical Services
JDC: American Jewish Joint Distribution Committee
JNC: Jewish National Council
UNRRA: United Nations Relief and Rehabilitation Administration
WWI: First World War
WWII: Second World War
